# ISUP grade upgrade prediction after radical prostatectomy: Role of Luteinizing Hormone to Testosterone ratio

**DOI:** 10.1002/bco2.70043

**Published:** 2025-07-14

**Authors:** Zhihua Pan, Ruizhe Zhao, Jinjiang Fan, Shaobo Zhang, Jie Li, Bianjiang Liu

**Affiliations:** ^1^ Department of Urology The First Affiliated Hospital of Nanjing Medical University Nanjing China; ^2^ Department of Urology Dongtai People's Hospital Dongtai Jiangsu China

**Keywords:** decision curve analysis, ISUP grade upgrading, logistic regression, luteinizing hormone to testosterone ratio, nomogram, prostate cancer, radical prostatectomy, ROC curve

## Abstract

**Purpose:**

This study aimed to evaluate the predictive value of the luteinizing hormone to testosterone (LH/T) ratio in postoperative International Society of Urological Pathology (ISUP) grade upgrading following radical prostatectomy.

**Materials and Methods:**

Clinical data from 503 patients who underwent radical prostatectomy (RP) at Jiangsu Provincial People's Hospital between June 2022 and October 2024 were collected. A stratified random sampling method was used to divide the patients into a training set and a validation set at a 7:3 ratio. In the training set, binary logistic regression analysis was applied to identify key predictive factors for postoperative ISUP classification upgrading. A nomogram predictive model and a multivariate forest plot were constructed. The validation set was assessed using the bootstrap method for C‐index, calibration curve, clinical impact curve (CIC) and decision curve analysis (DCA).

**Results:**

The postoperative ISUP upgrading rate was 31.2% (157/503). LH/T, Prostate Imaging Reporting and Data System (PI‐RADS) score, preoperative ISUP grade and biopsy method were identified as key predictors of pathological upgrading. The C‐index of the training set was 0.800, the validation set was 0.776, and the bootstrap resampling (500 times) in the validation set yielded a C‐index of 0.799, indicating high sensitivity and specificity in distinguishing different categories. Calibration curves demonstrated consistency between predicted and actual values, while clinical impact curve (CIC) and DCA confirmed the model's ability to optimize preoperative decision‐making.

**Conclusion:**

A lower LH/T ratio is associated with a higher risk of ISUP grade upgrading. As a novel predictive biomarker, LH/T may enhance preoperative risk assessment, aiding in more precise treatment decisions for prostate cancer patients.

## 1 INTRODUCTION

Prostate cancer has emerged as the most frequently diagnosed malignancy of the male genitourinary system worldwide.^1^ Radical prostatectomy (RP) remains the gold standard surgical approach for managing localized prostate cancer.^2^ However, despite preoperative prostate biopsy for pathological assessment, a subset of patients experiences an upgrade in their postoperative International Society of Urological Pathology (ISUP) grade group. This phenomenon, where the final histopathological evaluation reveals a higher malignancy grade than initially estimated via biopsy, can lead to inaccuracies in prognostic assessment and potentially influence clinical decision‐making.

The reported incidence of ISUP grade upgrading varies across different studies and institutions, with literature suggesting an approximate rate of 35%.^3^ In recent years, the widespread adoption of multiparametric magnetic resonance imaging (mpMRI) has significantly enhanced the anatomical delineation of the prostate and the characterization of malignant lesions.^4^ The combination of MRI‐ultrasound fusion‐targeted biopsy with systematic biopsy has markedly improved diagnostic accuracy and positive detection rates.^5^, ^6^ Nevertheless, despite these technological advancements, our high‐volume centre—where biopsy techniques are well‐established and quality control is rigorously maintained—continues to observe a substantial incidence of pathological upgrading, occurring in approximately 20%–30% of cases.

Given its potential implications for treatment planning and long‐term oncologic outcomes, accurately predicting postoperative ISUP grade upgrading has become a critical challenge in the optimization of prostate cancer management. Several studies have identified potential predictors of upgrading, including preoperative prostate‐specific antigen (PSA) levels, Prostate Imaging Reporting and Data System (PI‐RADS) scores and biopsy methodology. However, no universally accepted or highly accurate preoperative model currently exists to reliably predict ISUP upgrading.^7^, ^8^ Therefore, this study aims to evaluate the potential utility of the luteinizing hormone to testosterone (LH/T) ratio as a novel predictive biomarker for ISUP grade upgrading, with the ultimate goal of providing a more precise and clinically applicable risk stratification tool.

## 2 MATERIALS AND METHODS

### 2.1 Study population

**FIGURE 1:**
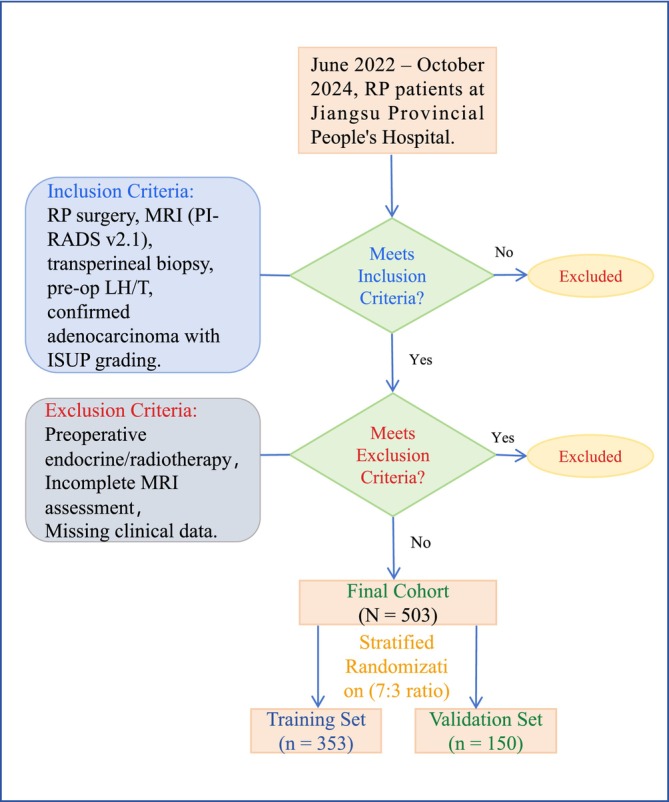
Patient grouping flow chart.

This study retrospectively collected data from patients who underwent RP at Jiangsu Provincial People's Hospital between June 2022 and October 2024. The inclusion criteria were as follows: (1) patients who underwent RP for prostate cancer; (2) preoperative MRI evaluation with PI‐RADS v2.1 scoring; (3) transperineal systematic biopsy, targeted biopsy or a combination of both, guided by cognitive fusion of MRI and ultrasound; (4) preoperative measurement of luteinizing hormone (LH) and testosterone (T) levels; and (5) histopathological confirmation of prostate adenocarcinoma with complete preoperative and postoperative ISUP grade data. Patients were excluded if they (1) had received androgen deprivation therapy (ADT), radiotherapy or chemotherapy prior to surgery; (2) had incomplete MRI assessments; or (3) had missing clinical data. After applying these criteria, a total of 503 eligible patients were selected. Using a stratified randomization approach, patients were divided into a training cohort (*n* = 353, 70%) and a validation cohort (*n* = 150, 30%), (Figure 1).

### 2.2 Data collection

Demographic and clinical characteristics, including age, height, weight, PSA levels and prostate volume, were collected for all patients. Body mass index (BMI) and prostate‐specific antigen density (PSAD) were calculated accordingly. PI‐RADS scores were obtained from preoperative multiparametric MRI scans. Hormonal parameters, including testosterone (T) levels, LH levels and the calculated LH/T ratio, were measured. Biochemical markers such as alkaline phosphatase (ALP) and lactate dehydrogenase (LDH) were also recorded. To minimize diurnal variations in hormone levels, blood samples were drawn in a fasting state between 07:00 and 09:00 on the morning following hospital admission. Biopsy techniques included systematic biopsy, targeted biopsy and a combination of both. The number of biopsy cores obtained via each method was documented. Histopathological parameters were assessed based on preoperative and postoperative Gleason scores, which were used to determine ISUP grade groups and evaluate the occurrence of ISUP grade upgrading.

### 2.3 Statistical analysis

All statistical analyses were conducted using SPSS version 22.0 and R software version 4.4.2. A two‐tailed *p*‐value <0.05 was considered statistically significant. Continuous variables were expressed as mean ± standard deviation (x̄ ± s) for normally distributed data and as median with interquartile range [M (Q1, Q3)] for nonnormally distributed data. Group comparisons were performed using independent samples *t*‐tests for normally distributed variables and Wilcoxon rank‐sum tests for nonnormally distributed variables. Categorical variables were presented as *n* (%) and compared using the chi‐square (χ^2^) test. In the training cohort, univariate logistic regression analysis was performed to evaluate potential predictors of postoperative ISUP grade upgrading, with variables achieving *p* < 0.2 being included in the multivariable logistic regression model. A stepwise backward selection approach, guided by the Akaike Information Criterion (AIC), was applied to identify independent predictors. A nomogram prediction model was constructed based on the identified predictors, and internal validation was conducted within the training cohort. The model's performance was assessed using: The concordance index (C‐index) to evaluate discriminative ability. Calibration curves to assess predictive consistency. Decision curve analysis (DCA) and clinical impact curves (CIC) to determine clinical applicability. For external validation, the model was tested in the validation cohort using 500 bootstrap resampling iterations to ensure robustness.

## 3 RESULTS

### 3.1 Baseline data analysis

The training cohort comprised **353 patients**, while the validation cohort included **150 patients**. Comparisons of **general characteristics, hormonal markers, biochemical parameters, biopsy methods and pathological indicators** between the two cohorts are presented in **Table** 1. No statistically significant differences were observed between the training and validation cohorts (*p* **> 0.05 for all variables**), indicating a well‐balanced distribution of baseline characteristics. The overall **postoperative ISUP grade upgrading rate was 31.2% (157/503)**, which is consistent with previously reported rates in both domestic and international studies.

**TABLE 1:** Baseline comparison of training and validation sets.

Variables	Total (*n* = 503)	Validation set (*n* = 150)	Training set (*n* = 353)	*p*
Group				0.439
Non‐Upgraded Group (*n*)	346 (69)	99 (66)	247 (70)	
Upgraded Group (*n*)	157 (31)	51 (34)	106 (30)	
Age (years)	69 (64, 74)	69 (63, 74)	69 (64, 74)	0.578
Weight (kg)	69 (64, 75)	70 (65, 75)	69 (63, 76)	0.606
Height (m)	1.7 (1.65, 1.73)	1.7 (1.65, 1.74)	1.7 (1.65, 1.73)	0.347
BMI (kg/m^2^)	24.44 ± 2.92	24.43 ± 2.75	24.44 ± 2.99	0.953
Follicle‐stimulating hormone (FSH, IU/L)	10.76 (7.16, 15.36)	11.21 (7.79, 15.03)	10.73 (6.85, 15.73)	0.423
Luteinizing hormone (LH, IU/L)	6.15 (4.47, 8.54)	6.07 (4.24, 8.38)	6.15 (4.6, 8.54)	0.670
Prolactin (mIU/L)	253.28 (196, 333.44)	256.74 (194.61, 343.81)	252.37 (198.04, 331.74)	0.969
Estradiol (pmol/L)	90.78 (73.06, 109.3)	91.03 (73.81, 110.61)	90.6 (72.7, 109.17)	0.977
Testosterone (nmol/L)	12.51 (9.93, 15.13)	12.54 (9.93, 15.11)	12.49 (9.94, 15.12)	0.884
LH/T Ratio (IU/nmol)	0.49 (0.35, 0.7)	0.48 (0.34, 0.69)	0.49 (0.36, 0.7)	0.775
T/LH Ratio (nmol/IU)	2.05 (1.44, 2.87)	2.08 (1.46, 2.94)	2.04 (1.44, 2.78)	0.782
Alkaline phosphatase (ALP, U/L)	69 (59, 81)	68 (58, 81.75)	70 (60, 81)	0.342
Lactate dehydrogenase (LDH, U/L)	178 (157, 202)	178 (160.25, 201.5)	178 (156, 202)	0.502
Free PSA (fPSA, ng/mL)	1.33 (0.85, 2.07)	1.32 (0.84, 2.11)	1.33 (0.86, 2.05)	0.625
Total PSA (tPSA, ng/mL)	13.81 (9.14, 23.64)	13.03 (9.86, 21.49)	14.2 (8.9, 25.74)	0.473
fPSA/tPSA Ratio	0.09 (0.07, 0.13)	0.09 (0.07, 0.13)	0.09 (0.07, 0.13)	0.467
Prostate volume (mL)	34.05 (27.12, 46.59)	34.06 (26.85, 47.82)	34.05 (27.16, 46.48)	0.663
PSA density (PSAD, ng/mL/mL)	0.41 (0.26, 0.72)	0.4 (0.25, 0.66)	0.42 (0.26, 0.74)	0.215
PI‐RADS score	4 (4, 5)	4 (4, 5)	4 (4, 5)	0.471
Suspicious metastatic lesion				0.362
Yes	312 (62)	88 (59)	224 (63)	
No	191 (38)	62 (41)	129 (37)	
Target located in MRI peripheral zone				0.621
Yes	123 (24)	34 (23)	89 (25)	
No	380 (76)	116 (77)	264 (75)	
Target located in MRI Non‐peripheral zone				0.867
Yes	246 (49)	72 (48)	174 (49)	
No	257 (51)	78 (52)	179 (51)	
Systematic biopsy core count	12 (12, 12)	12 (12, 12)	12 (12, 12)	0.632
Targeted biopsy core count	4 (4, 4)	4 (1, 4)	4 (4, 6)	0.429
Total biopsy core count	16 (12, 16)	16 (12, 16)	16 (12, 16)	0.962
Total positive biopsy core count	6 (4, 8)	6 (4, 9)	6 (4, 8)	0.260
Total biopsy positivity rate	0.4 (0.25, 0.58)	0.44 (0.25, 0.63)	0.38 (0.25, 0.56)	0.355
Biopsy method				0.411
1 (Systematic biopsy)	117 (23)	38 (25)	79 (22)	
2 (Targeted biopsy)	34 (7)	7 (5)	27 (8)	
3 (Combined systematic and targeted biopsy)	352 (70)	105 (70)	247 (70)	
Interval between biopsy and radical prostatectomy (days)	11 (7, 15)	11 (8, 15)	11 (7, 15)	0.387
Preoperative ISUP grade	2 (2, 3)	2 (2, 3)	2 (2, 3)	0.549
Postoperative ISUP grade	3 (2, 3)	3 (2, 3)	3 (2, 3)	0.555

*Note*: LH/T represents the ratio of luteinizing hormone (LH) to testosterone (T); T/LH represents the ratio of testosterone (T) to luteinizing hormone (LH).

### 3.2 Univariate analysis

In the training cohort, univariate logistic regression analysis was performed to calculate the *p*‐values for each variable (Table 2). Biopsy method was treated as a dummy variable. Notably, LH showed a *p*‐value of 0.019, while LH/T and T/LH exhibited even lower *p*‐values, suggesting a stronger association.

**TABLE 2:** Univariate logistic regression analysis.

Variables	B	SE	OR(95%CI)	Z	*p*
Preoperative ISUP grade	−0.843	0.13391	0.431(0.328–0.555)	−6.294	<0.001
Interval between biopsy and radical prostatectomy (days)	0.011	0.0061	1.012(1–1.025)	1.885	0.059
Biopsy Method 2 (targeted biopsy)	−0.515	0.46658	0.597(0.231–1.462)	−1.105	0.269
Biopsy Method 3 (combined systematic and targeted biopsy)	−0.937	0.26982	0.392(0.231–0.666)	−3.473	0.001
Total biopsy positivity rate	−0.567	0.48053	0.567(0.217–1.435)	−1.181	0.238
Total positive biopsy core count	−0.064	0.03426	0.938(0.876–1.002)	−1.859	0.063
Total biopsy core count	−0.052	0.03138	0.949(0.892–1.01)	−1.653	0.098
Targeted biopsy core count	−0.088	0.04216	0.916(0.842–0.994)	−2.085	0.037
Systematic biopsy core count	−0.001	0.03458	0.999(0.935–1.072)	−0.041	0.967
Target located in MRI non‐peripheral zone	0.284	0.23331	1.329(0.842–2.105)	1.218	0.223
Target located in MRI peripheral zone	0.199	0.27339	1.22(0.721–2.114)	0.728	0.467
Suspicious metastatic lesions	0.245	0.23868	1.278(0.798–2.037)	1.027	0.304
PI‐RADS score	0.28	0.1371	1.323(1.019–1.747)	2.044	0.041
PSA density (PSAD, ng/mL/mL)	0.004	0.21357	1.004(0.642–1.508)	0.02	0.984
Prostate volume (mL)	0.008	0.00596	1.008(0.996–1.02)	1.319	0.187
Total PSA (tPSA, ng/mL)	0	0.00683	1(0.986–1.013)	0.011	0.992
Free PSA (fPSA, ng/mL)	0.098	0.06659	1.103(0.967–1.26)	1.477	0.140
Lactate dehydrogenase (LDH, U/L)	−0.002	0.00343	0.998(0.991–1.004)	−0.662	0.508
Alkaline phosphatase (ALP, U/L)	−0.004	0.00653	0.996(0.983–1.008)	−0.644	0.519
T/LH ratio (nmol/IU)	0.249	0.08639	1.282(1.085–1.526)	2.878	0.004
LH/T ratio (IU/nmol)	−1.095	0.40262	0.335(0.143–0.695)	−2.719	0.007
Testosterone (nmol/L)	0.035	0.02759	1.036(0.981–1.094)	1.282	0.200
Estradiol (pmol/L)	0.007	0.00376	1.007(1–1.015)	1.912	0.056
Prolactin (mIU/L)	0	0.00045	1(0.999–1.001)	−0.055	0.956
Luteinizing hormone (LH, IU/L)	−0.086	0.03649	0.918(0.851–0.983)	−2.347	0.019
Follicle‐stimulating hormone (FSH, IU/L)	−0.042	0.01571	0.959(0.928–0.987)	−2.654	0.008
BMI (kg/m^2^)	0.017	0.03892	1.017(0.942–1.098)	0.441	0.659
Height (m)	0.218	2.10107	1.244(0.02–77.13)	0.104	0.917
Weight (kg)	0.005	0.01179	1.005(0.982–1.029)	0.457	0.648
Age (years)	−0.027	0.01647	0.974(0.942–1.005)	−1.631	0.103

*Note*: The biopsy method has been treated as a dummy variable, including (1) systematic biopsy group, (2) targeted biopsy group and (3) combined systematic and targeted biopsy group, with the systematic biopsy group serving as the reference. The *p*‐values of LH/T and T/LH are significantly lower than that of luteinizing hormone.

### 3.3 Multivariate logistic regression analysis and prediction model construction

**FIGURE 2:**
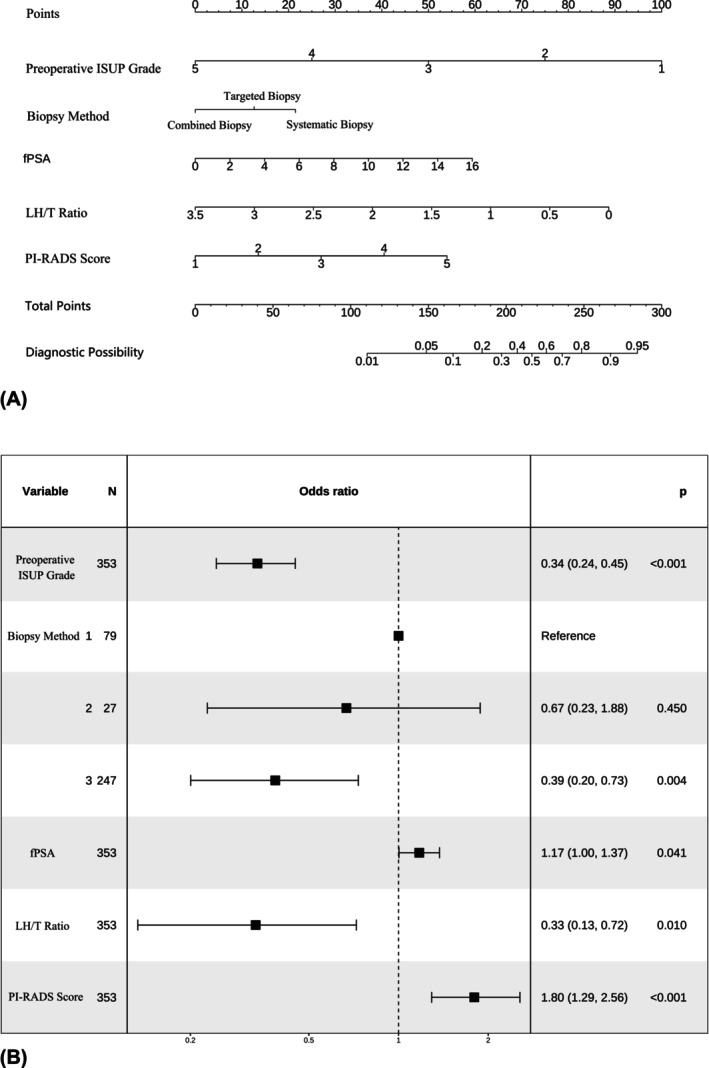
(A) Nomogram. (B) Multivariate regression Forest plot (Biopsy Method: (1) Systematic, (2) Targeted, (3) Combined; Reference: 1)

During the selection process, variables with *p* < 0.2 in the univariate logistic regression analysis were included in the multivariate logistic regression model. A stepwise backward selection method was applied, with AIC used to identify independent predictors of postoperative ISUP grade upgrading (Table 3). The results showed that fPSA, LH/T, PI‐RADS score, preoperative ISUP grade and Biopsy Method 3 were significantly associated with ISUP upgrading (*p* < 0.05). Although Biopsy Method 2 had a *p*‐value >0.05, it was retained in the model based on the principle that dummy variables should be entered and removed simultaneously, ensuring better model interpretability and robustness. A nomogram and a multivariate regression forest plot were constructed based on these predictors (Figure 2A,B). The preoperative ISUP grade, Biopsy Method 3, fPSA, LH/T and PI‐RADS score were identified as key predictors of postoperative pathological upgrading in prostate cancer patients. Among these, preoperative ISUP grade, Biopsy Method 3 and LH/T acted as negative predictors, while the others were positive predictors.

**TABLE 3:** Multivariate logistic regression analysis.

Variables	B	SE	OR(95%CI)	Z	*p*
Intercept	0.261	0.79927	1.298(0.266–6.182)	0.327	0.744
Preoperative ISUP grade	−1.091	0.15538	0.335(0.244–0.450)	−7.021	<0.001
Biopsy Method 2	−0.403	0.53411	0.668(0.228–1.879)	−0.755	0.450
Biopsy Method 3	−0.953	0.32948	0.385(0.200–0.732)	−2.892	0.004
fPSA (ng/mL)	0.16	0.07842	1.173(1.003–1.372)	2.043	0.041
LH/T Ratio (IU/nmol)	−1.105	0.42754	0.331(0.133–0.721)	−2.583	0.010
PI‐RADS score	0.585	0.1737	1.795(1.291–2.555)	3.37	0.001

*Note*: The *p*‐value of LH/T is 0.01, indicating significant statistical relevance and a substantial contribution to the model. The variance inflation factor (VIF) values for all independent variables were close to 1, indicating the absence of multicollinearity.

### 3.4 Prediction model evaluation

**FIGURE 3:**
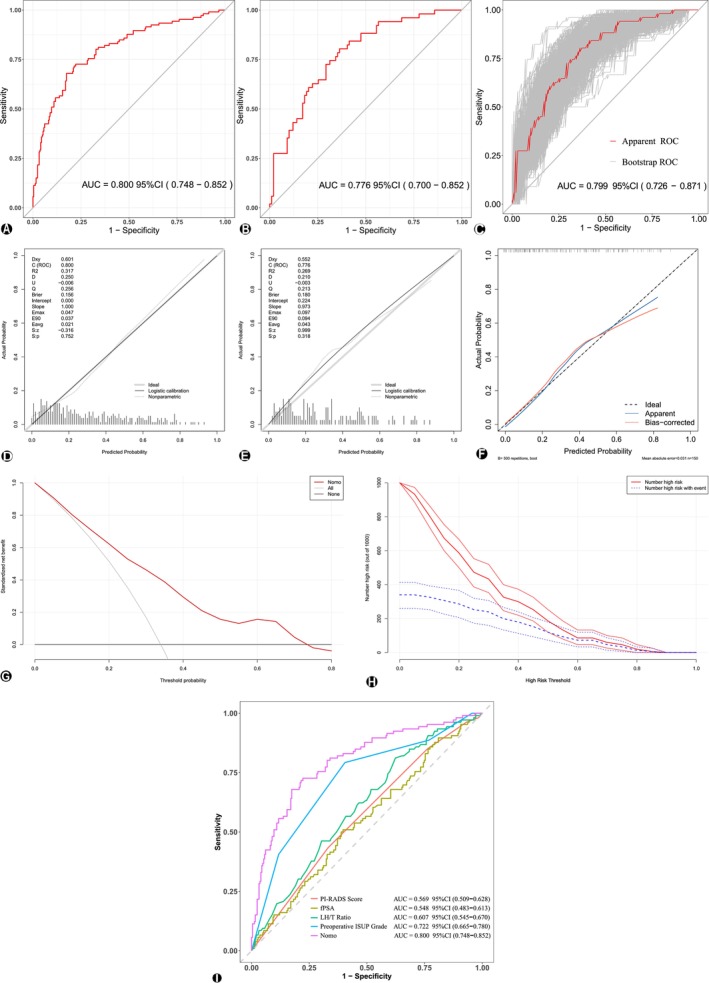
(A,B,C) **Receiver operating characteristic (ROC) curve: (**A) Training set; (B) Validation set; (C) Bootstrap resampling of validation set (500 iterations). (D,E,F) **Calibration curves for training and validation sets: (**D) Training set; (E) validation set; (F) bootstrap resampling of validation set (500 iterations). (G,H) **Clinical evaluation of the predictive model:** (G) Decision curve analysis (DCA) for validation set with bootstrap resampling (500 iterations); (H) clinical impact curve (CIC) for validation set with bootstrap resampling (500 iterations). (I) **ROC curves of the model and individual predictors in the training set.** The model showed the highest AUC.

The C‐index for the training cohort was 0.800, while that for the validation cohort was 0.776. After 500 bootstrap resampling iterations, the validation cohort C‐index reached 0.799 (95% CI: 0.726–0.871), indicating good discriminative ability (Figure 3A,B,C). Calibration curves for the training cohort, validation cohort and bootstrap validation (500 resampling iterations) demonstrated good agreement between predicted and observed outcomes (Figure 3D,E,F). The Hosmer–Lemeshow test yielded *p*‐values of 0.338 and 0.430 for the training and validation cohorts, respectively, both well above 0.05, confirming good model fit with no significant deviation from expectations. In the clinical DCA, the net benefit range was observed between 0.03 and 0.74 in the validation cohort, supporting clinical utility of the model (Figure 3G). The CIC for the training cohort showed convergence at 0.34, further reinforcing the model's clinical applicability (Figure 3H). In the ROC analysis based on predicted probabilities in the training set, the current model demonstrated the highest AUC compared to individual predictors, suggesting superior discriminative ability and model robustness (Figure 3I).

## 4 DISCUSSION

Postoperative ISUP grade upgrading remains a critical issue in the clinical management of prostate cancer.^9^ Due to the multifocal and heterogeneous nature of prostate cancer,^10^, ^11^ one of the most common reasons for pathological upgrading is the widespread use of ultrasound‐guided systematic biopsy, which primarily delineates the prostate's contour and benign nodules. This approach is inherently blind, often missing prostate lesions, particularly those with the highest malignancy. Moreover, the experience of the performing physician and variations in biopsy equipment further limits the accuracy of pathological grading.

Although mpMRI and MRI‐ultrasound fusion‐targeted biopsy have significantly improved biopsy accuracy and cancer detection rates, the risk of pathological upgrading remains. Developing more precise predictive models and optimizing treatment strategies are key challenges in the clinical management of prostate cancer.

### 4.1 The role of sex hormones in prostate cancer

Sex hormones play a pivotal role in the initiation, progression and metastasis of prostate cancer.^12^, ^13^ Androgens such as testosterone (T) bind to androgen receptors (AR) on the cell membrane, mediating a cascade of cellular functions and gene expression.^14^, ^15^ The prostate is a primary target organ for testosterone, where 5α‐reductase converts testosterone into dihydrotestosterone (DHT), a more potent androgen that regulates prostate cell proliferation and differentiation.^16^, ^17^ Abnormal testosterone levels may lead to malignant transformation of prostate cells, and elevated testosterone is significantly associated with the development of advanced prostate cancer.

LH stimulates testicular testosterone production, further promoting prostate cancer cell proliferation.^17^ Multiple studies have demonstrated that LH levels are typically elevated in prostate cancer patients compared to healthy controls,^18^, ^19^, ^20^ and LH levels correlate with both the risk and aggressiveness of prostate cancer. Therefore, we hypothesize that sex hormones may be a key factor in ISUP grade upgrading.^21^

### 4.2 LH/T as a novel predictor of pathological upgrading

To date, no studies have incorporated sex hormones as a key factor in predicting postoperative pathological upgrading. Our model revealed that for every unit increase in the LH/T ratio, the likelihood of postoperative ISUP grade upgrading decreased by approximately 66.9%, indicating a protective effect. In other words, a lower amount of LH relative to testosterone corresponds to a higher risk of upgrading. This suggests an imbalance in the role of LH in testosterone regulation.^22^, ^23^, ^24^

When LH/T is high, prostate cancer cells may remain relatively stable, making further deterioration and pathological upgrading less likely. Conversely, when LH/T is low, the T/LH ratio increases, meaning that each unit of LH corresponds to a higher level of testosterone, which may destabilize prostate cancer cells, leading to further malignant progression and pathological upgrading. Notably, traditional testosterone measurements often remain within the normal range, making them ineffective predictors of pathological upgrading. This underscores the clinical significance of LH/T in our study.

Initially, we considered incorporating T/LH into the model, but LH/T was ultimately selected based on the lowest AIC. However, both ratios demonstrated clinical value.

### 4.3 Biopsy method and its impact on pathological upgrading

Our model incorporated biopsy method, preoperative ISUP grade, PI‐RADS score and fPSA levels to enhance predictive accuracy. We found that Biopsy Method 2 (targeted biopsy) did not significantly differ from systematic biopsy in predicting pathological upgrading.^25^, ^26^ This finding highlights the impact of prostate cancer's heterogeneous distribution on biopsy outcomes.^27^

However, Biopsy Method 3 (combined systematic and targeted biopsy) significantly reduced sampling errors and decreased the risk of upgrading.^28^ Additionally, lower preoperative ISUP grades, higher PI‐RADS scores and elevated fPSA levels were significant predictors of postoperative upgrading.

### 4.4 Clinical implications of the model

Our nomogram‐based predictive model enables clinicians to assess the risk of pathological upgrading at the biopsy stage, facilitating more informed treatment decisions.

For instance, ISUP grade 1 (Gleason score ≤6) is often considered clinically insignificant prostate cancer. If a patient is at low risk of upgrading, surgery, radiotherapy or endocrine therapy may not be necessary, and active surveillance could be a viable alternative. This approach minimizes treatment‐related side effects and preserves quality of life, particularly in elderly or comorbid patients.^29^

Conversely, ISUP grade ≥2 (Gleason score >7) represents clinically significant prostate cancer. For patients with a high risk of upgrading, aggressive intervention may be warranted. In non‐surgical candidates or those requiring neoadjuvant therapy, intensified treatment strategies—such as oral next‐generation androgen receptor inhibitors—could be considered. For surgical patients, extended pelvic lymph node dissection may improve outcomes and optimize oncologic control.

By reducing overtreatment of low‐risk patients and undertreatment of high‐risk patients, this model may enhance risk stratification, minimize patient burden, optimize healthcare resource utilization and improve clinical outcomes.

## 5 CONCLUSIONS

This study is the first to identify a significant association between a lower LH/T ratio and postoperative ISUP grade upgrading (*p* < 0.05), suggesting LH/T as a novel predictive biomarker. A low LH/T ratio (or a high T/LH ratio) may indicate an increased risk of pathological upgrading, further reinforcing the impact of androgen axis dysregulation on tumour biology.^30^

However, the underlying mechanisms by which LH/T influences tumour progression remain to be fully elucidated. Further multicenter validation is necessary to enhance the model's accuracy, stability and clinical applicability.

## AUTHOR CONTRIBUTIONS

Liu **Bianjiang and Pan Zhihua**: Study design and manuscript writing. **Pan Zhihua, Zhao Ruizhe and Zhang Shaobo**: Data analysis and visualization. **Pan Zhihua and Fan Jinjiang**: Data processing. **Li Jie and Liu Bianjiang**: Research supervision and manuscript review.

## CONFLICT OF INTEREST STATEMENT

All authors declare no conflicts of interest.

## Data Availability

The data supporting the findings of this study are not publicly available due to patient confidentiality and institutional restrictions but can be obtained from the corresponding author upon reasonable request.
